# Safety and Efficacy of Direct Oral Anticoagulants Versus Standard Therapy for Venous Thromboembolism in Cancer Patients: A Systematic Review and Network Meta-Analysis of Randomized Clinical Trials

**DOI:** 10.3390/jcm15031090

**Published:** 2026-01-30

**Authors:** Alaa Shahbar, Sabah Alshahrani, Abdullah Alhifany, Mohammed Alnuhait, Afnan Noor, Abdulaali Almutairi, Faisal A. Alhamdan, Ahmad A. Alshamrani, Alqassem Y. Hakami

**Affiliations:** 1 Pharmacy Practices Department, College of Pharmacy, Umm Al-Qura University, Taif Road, Alabdiah, Makkah 21955, Saudi Arabia; 2 Asir Health Cluster, Asir Central Hospital, Clinical Pharmacy Department, Abha 62523, Saudi Arabia; 3 Department of Clinical Pharmacy, College of Pharmacy, Shaqra University, Al-Dawadmi Campus, Al-Dawadmi 11961, Saudi Arabia; 4 Pharmaceutical Care Services Department, King Faisal Specialist Hospital and Research Center, Jeddah 23433, Saudi Arabia; 5 Department of Pharmacy Practice, College of Pharmacy, Alfaisal University, Riyadh 11533, Saudi Arabia; 6 King Abdullah International Medical Research Center, Jeddah 22384, Saudi Arabia; 7 College of Medicine, King Saud Bin Abdulaziz University for Health Sciences, Jeddah 21423, Saudi Arabia; 8 Pharmaceutical Care Department, Ministry of the National Guard—Health Affairs, Jeddah 11426, Saudi Arabia

**Keywords:** cancer, direct oral anticoagulants, low molecular weight heparin, warfarin, VTE recurrence, major bleeding, CRNMB

## Abstract

**Background**: Patients with malignancy demonstrate an elevated risk of developing venous thromboembolism (VTE) due to coagulopathy and treatment modalities. Although low-molecular-weight heparin (LMWH) and warfarin have historically been standard therapies, direct oral anticoagulants (DOACs) are increasingly used in this population. **Methods**: We conducted a Bayesian network meta-analysis to compare the safety and efficacy of apixaban, edoxaban, rivaroxaban, LMWH, and warfarin in cancer-associated VTE. A comprehensive literature search of Embase, Medline, clinical trial registries, and manual sources was performed up to November 2025. The primary outcome was to compare the risk of VTE recurrence across therapies. Secondary outcomes included major bleeding, clinically relevant non-major bleeding (CRNMB), and all-cause mortality. Odds ratios (ORs) with 95% credible intervals (CrIs) were estimated, and treatment rankings were derived using the surface under the cumulative ranking curves (SUCRA) probabilities. **Results**: Seven randomized controlled trials (RCTs) involving 3325 patients were included. No clear evidence of differences was observed among apixaban, rivaroxaban, edoxaban, low-molecular-weight heparin, and warfarin for VTE recurrence, major bleeding, clinically relevant non-major bleeding, and mortality. For instance, rivaroxaban showed no statistically significant difference in VTE recurrence compared with apixaban (OR 1.13; 95% CrI 0.17–12.27), warfarin (OR 0.60; 95% CrI 0.03–19.00), edoxaban (OR 0.77; 95% CrI 0.06–11.11), or LMWH (OR 0.51; 95% CrI 0.10–2.66). Wide credible intervals reflect uncertainty due to the limited number of RCTs and low event rates. **Conclusions**: This Bayesian network meta-analysis showed no statistically significant differences between therapies with respect to VTE recurrence, bleeding outcomes, and mortality. However, the wide credible intervals indicate limited precision, warranting cautious interpretation of the findings.

## 1. Introduction

Oncology patients face an increased risk of morbidity and mortality due to cancer-associated thrombosis, a complication that has become more prevalent over time. Pulmonary embolism (PE) and deep vein thrombosis (DVT), which are the two primary forms of venous thromboembolism (VTE), are well-documented complications in cancer patients, significantly reducing overall survival rates [1].

Managing VTE in cancer patients presents unique challenges, necessitating treatment strategies that differ from conventional approaches [2]. Historically, the management of acute VTE begins with the administration of a fast-acting injectable anticoagulant, such as low-molecular-weight heparin (LMWH), fondaparinux, or unfractionated heparin, followed by warfarin. However, this regimen poses significant difficulties in cancer patients due to frequent drug–drug interactions (DDIs) with warfarin, which can either amplify or diminish their anticoagulant effects. As a result, LMWH has emerged as a preferred anticoagulation therapy for cancer-associated VTE due to its reliable mechanism of action [3].

DOACs (Direct Oral Anticoagulants) were developed to surmount the limitations of warfarin, which exhibit unpredictable pharmacokinetics and pharmacodynamics by inhibiting the liver’s vitamin K-dependent synthesis of clotting factors II, VII, IX, and X. The DOAC class comprises both direct factor Xa inhibitors—rivaroxaban, apixaban, and edoxaban—and the direct thrombin (factor IIa) inhibitor dabigatran [4,5].

Rivaroxaban, apixaban, and edoxaban offer several advantages over warfarin, including a more predictable anticoagulant effect by directly inhibiting factor Xa, fixed dosing, and fewer drug–drug interactions. Furthermore, their use has expanded to include patients with cancer-associated thrombosis (CAT), offering an alternative therapeutic option for this high-risk population [6].

Over the past decade, rivaroxaban, apixaban, and edoxaban have emerged as alternative treatment options for cancer-associated VTE. Randomized controlled trials comparing them with LMWH have demonstrated non-inferior efficacy for the prevention of recurrent VTE, with variable safety profiles, particularly concerning bleeding risk. Clinical trials including AMPLIFY, ADAM-VTE, CARAVAGGIO, SELECT-D, CASTA-DIVA, Hokusai VTE Cancer, and the study by Mokadem et al. (2020) have provided evidence supporting the use of apixaban, rivaroxaban, and edoxaban in patients with cancer-associated VTE [7,8,9,10,11,12,13]. These findings go along with a systematic review and meta-analysis, which reported no statistically significant difference in VTE recurrence at 6 months between DOACs and LMWH (relative risk [RR]: 0.65, 95% CI: 0.42–1.01). However, the study found a significantly higher risk of major bleeding associated with DOACs (RR: 1.74, 95% CI: 1.05–2.88) [14].

However, the factor IIa inhibitor, dabigatran, efficacy and safety outcomes for specifically VTE patients with cancer were not studied in randomized controlled trials. Although the RE-COVER I and RE-COVER II trials enrolled a limited number of patients with active malignancy, these studies did not provide cancer-specific analyses for key outcomes such as VTE recurrence, major bleeding, clinically relevant non-major bleeding, or mortality. Consequently, the available data were insufficient to support a robust and reliable assessment of dabigatran in this patient population [15,16,17].

This study aimed to evaluate the efficacy and safety of apixaban, rivaroxaban, and edoxaban using a Bayesian network meta-analysis of randomized controlled trials involving patients with cancer-associated venous thromboembolism. By integrating both direct and indirect evidence, the network meta-analytic approach enables a comparative assessment among direct oral anticoagulants in the absence of head-to-head trials and overcomes the limitations inherent to conventional pairwise meta-analyses, which are restricted to two treatment comparisons.

## 2. Methods

### 2.1. Search Strategy

We systematically searched Embase, Medline, and ClinicalTrials.gov, and manual searches covering studies until November 2025. The search strategy employed a combination of relevant keywords, including “Anticoagulants,” “Cancer,” “Tumor,” “Apixaban,” “Edoxaban,” “Rivaroxaban,” “Low-Molecular-Weight Heparin,” “Venous Thromboembolism,” “Pulmonary Embolism,” “Venous Thrombosis,” “Randomized Controlled Trial,” and “Controlled Clinical Trial.” To supplement the database search, we manually reviewed the bibliographies of retrieved articles and relevant review papers to identify additional studies. The screening process involved examining the titles and abstracts of identified studies to ensure they met our predefined inclusion criteria. Each screening step was independently performed by two reviewers, with the initial selection based on titles followed by an evaluation of abstracts. In cases of disagreement, a third reviewer was consulted to resolve conflicts and reach a consensus regarding the inclusion of studies. This review was conducted in accordance with the PRISMA guidelines but was not prospectively registered. PRISMA checklist see the Supplementary Materials.

### 2.2. Study Selection

Eligible studies were randomized controlled trials (RCTs) conducted in adult patients (≥18 years) with histologically or clinically confirmed active malignancy who were diagnosed with venous thromboembolism (VTE), including deep vein thrombosis (DVT), pulmonary embolism (PE), or both. Studies were required to evaluate the therapeutic use of direct oral anticoagulants (DOACs)—including apixaban, rivaroxaban, or edoxaban—administered for the treatment of cancer-associated VTE and to include a comparator arm consisting of low-molecular-weight heparin (LMWH) or warfarin.

To be eligible for inclusion, studies were required to report at least one of the following predefined outcomes: (1) objectively confirmed recurrent VTE, (2) major bleeding, (3) clinically relevant non-major bleeding (CRNMB), or (4) all-cause mortality. When multiple publications derived from the same clinical trial were identified, only the most comprehensive report with the longest available follow-up was included to avoid data duplication.

Studies were excluded if they were non-randomized in design (e.g., observational studies, case–control studies, registry analyses), involved pediatric populations, or enrolled non-cancer patients. Trials evaluating anticoagulation regimens without a DOAC comparator, studies lacking extractable outcome data for the predefined endpoints, and publications without cancer-specific analyses were also excluded. In addition, conference abstracts, case reports, review articles, and studies with insufficient or incomplete outcome reporting were excluded to ensure methodological rigor and analytic validity.

### 2.3. Study Outcomes

The meta-analysis primarily evaluated the incidence of recurrent venous thromboembolism (VTE). Secondary outcomes included safety endpoints such as major bleeding, clinically relevant non-major bleeding (CRNMB), and overall mortality from any cause.

### 2.4. Data Extraction and Quality Assessment

We independently collected the following data for each study: study design, participant characteristics, intervention details, and outcome measures. Participant characteristics included the total number of participants, age, gender, and whether they had active or metastatic cancer at enrollment. Intervention specifics involved the type of anticoagulant used, dosage, duration, and frequency of administration. Finally, the outcome measures included the primary and secondary outcomes, along with the number of patients in each treatment group who experienced these outcomes.

### 2.5. Study Risk of Bias Assessment

To evaluate the reliability of the eligible randomized trials, two reviewers independently assessed critical aspects of the trial methodology using the Cochrane Risk of Bias tool. The areas assessed included selection sequence generation, allocation concealment, blinding of participants and personnel, blinding of outcome assessment, completeness of outcome data, selective reporting, and any additional potential biases. The risk of bias for each study was categorized as high, medium, or low. Any disagreements with the reviewer were resolved through consultation with experienced authors or discussion until a consensus was reached.

### 2.6. Statistical Analysis

We conducted a Bayesian network meta-analysis using MetaInsight, an online platform integrating R packages (V6.0.0) to execute network meta-analyses. Given the few included studies, we selected the fixed-effect model, which provided a posterior mean of the residual deviance score comparable to that of the random-effect model. For each outcome, the odds ratio and 95% credible interval were estimated using Markov chain Monte Carlo techniques over 25,000 iterations. Convergence of the model was assessed using Gelman-Rubin plots to ensure accurate results. The relative ranking of treatments for each outcome was determined by calculating the surface under the cumulative ranking curves (SUCRA) and analyzing the distribution of ranking probabilities.

## 3. Results

**Figure 1:**
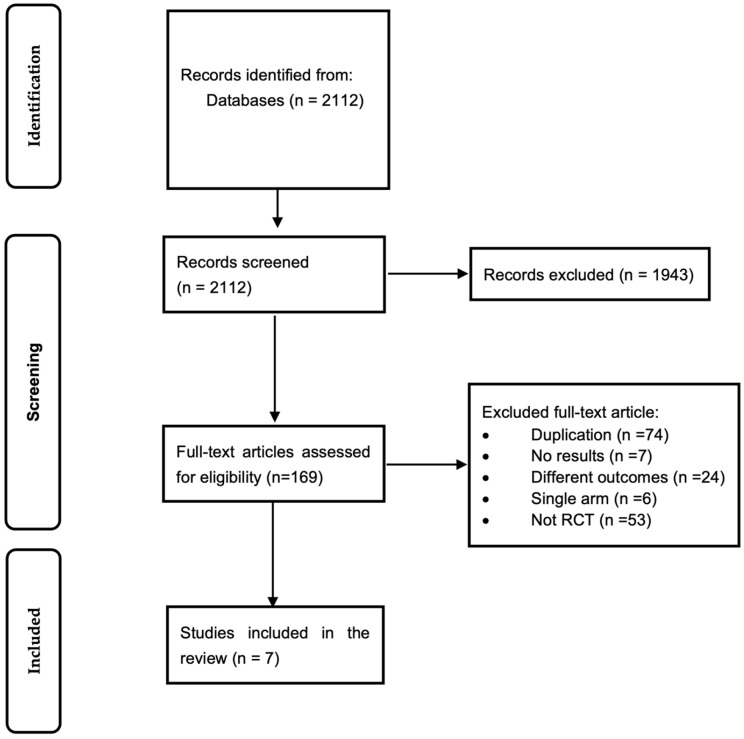
Preferred Reporting Items Meta-Analyses (PRISMA) flow diagram.

The literature search conducted as of November 2025 resulted in a total of 2112 citations. After the eligibility assessment, 1943 articles were excluded for not meeting the criteria, leaving 169 articles for further review. Of these, 162 were excluded for specific reasons: 74 were duplicates, 7 had no results, 24 presented different outcomes, and 6 were single-arm studies rather than randomized controlled trials (RCTs). After a thorough review, seven RCTs, including 3325 participants, were included in the meta-analysis. Figure 1 provides a schematic overview of the literature search process, while Table 1 summarizes the participants’ characteristics.

The follow-up duration was 6 months for five studies, and 3 months and 12 months for the remaining two studies, respectively. Four studies used apixaban, two studies used rivaroxaban, and one utilized edoxaban. No studies included dabigatran although the term DOACs (Direct Oral Anticoagulants) encompasses Apixaban, Edoxaban, Rivaroxaban, and Dabigatran. The comparator treatments in the seven studies were LMWH or warfarin.

### 3.1. Quality Assessment

**Figure 2:**
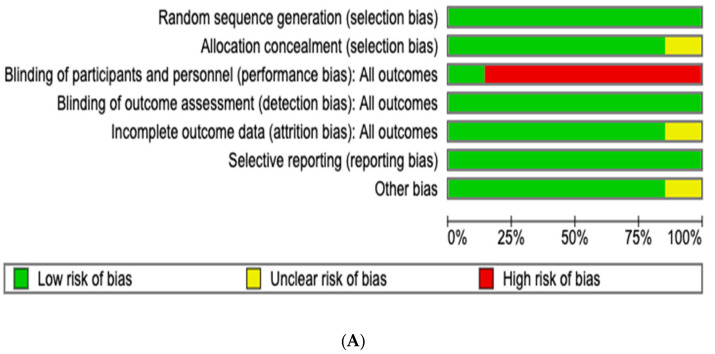

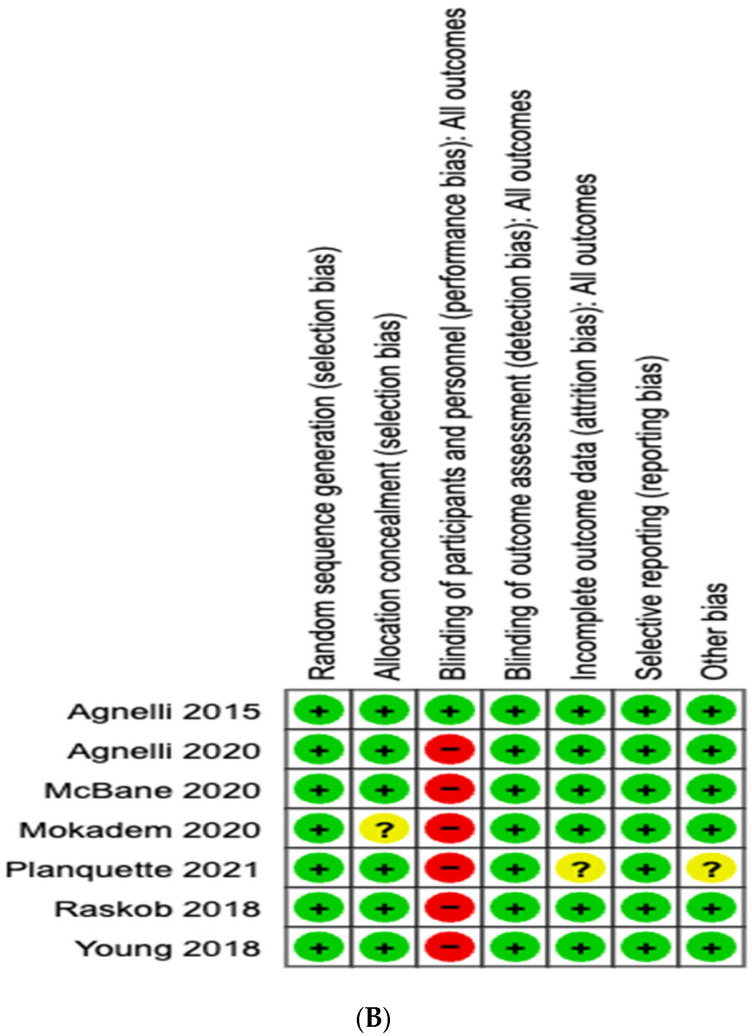
Risk of bias of 7 randomized controlled trials (RCTs) included in the meta-analysis. (**A**) The risk of bias graph reviews the authors’ judgments about each risk of bias item presented as percentages across all included studies. (**B**) Risk of bias summary reviews the authors’ judgments about each risk of bias item for each included study. Green (+) indicates low risk of bias, yellow (?) indicates some concerns, and red (−) indicates high risk of bias.

The risk of bias assessment for the included studies in the meta-analysis demonstrated a generally low risk across most key domains, supporting the overall reliability of the findings. All trials followed appropriate randomization procedures and maintained adequate allocation concealment, minimizing selection bias risk. However, the central area of concern was performance bias. Agnelli 2020, McBane 2020, Young 2018, Planquette 2021 and Raskob 2018 were open-label trials, which inherently carry a high risk of performance bias as participants and investigators were aware of the treatment allocations [7,8,10,12,13]. Additionally, Mokadem 2020 employed a single-masked design, where participants were blinded, but the investigators were not, further contributing to performance bias in this study. Moreover, Mokadem 2020 presented an unclear risk of detection bias due to insufficient details regarding the blinding of outcome assessments [11]. The trial by Planquette et al. (2021) [12] did not reach its planned sample size because of slow patient enrollment, which led to early termination. In addition, approximately 9% of participants were lost to follow-up and were censored at their last recorded assessment. The quality assessment results are summarized in Figure 2.

### 3.2. VTE Recurrence

**Figure 3:**
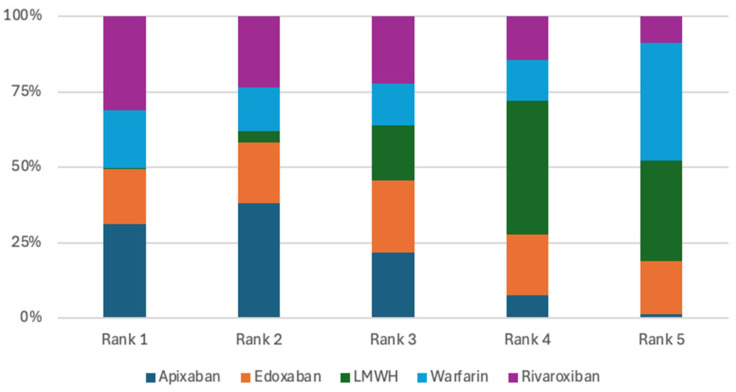
Treatments ranking for VTE recurrence.

Seven randomized controlled trials were included in the analysis of VTE recurrence [7,8,9,10,11,12,13]. Across these studies, there was no evidence that apixaban, rivaroxaban, edoxaban, or warfarin significantly reduced the risk of recurrent venous thromboembolism compared with low-molecular-weight heparin (LMWH) or when compared against each other. (Table 2). Apixaban ranked highest in terms of probability for reducing the risk of VTE recurrence, with a 31.07% probability of being the most effective treatment option, closely followed by rivaroxaban at 31.06% (Figure 3). This finding indicates that apixaban and rivaroxaban had the highest ranking probabilities for VTE recurrence; however, these rankings represent probabilistic estimates and should not be interpreted as evidence of clinical superiority.

### 3.3. Major Bleeding

**Figure 4:**
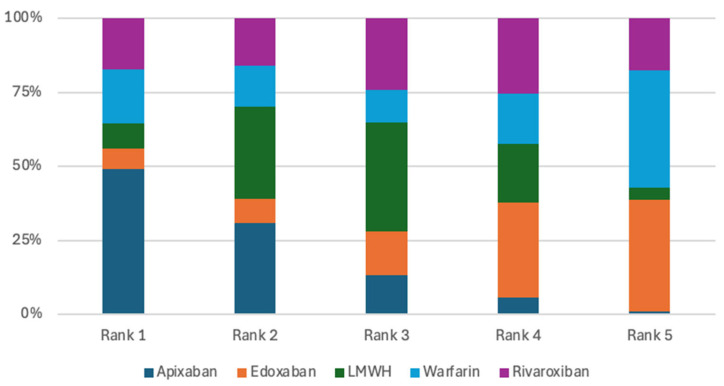
Treatments ranking for major bleeding.

The primary bleeding analysis included seven studies [7,8,9,10,11,12,13]. Pairwise comparisons among the evaluated treatments showed no statistically significant differences in their ability to reduce major bleeding (Table 3). Nevertheless, apixaban had the highest probability of being ranked first for minimizing major bleeding, with a 49.26% likelihood of occupying the top position (Figure 4). Although apixaban was ranked highest in terms of minimizing major bleeding, this ranking reflects a probabilistic estimate rather than a true treatment effect due to the wide credible intervals and low event rates.

### 3.4. Clinically Relevant Non-Major Bleeding (CRNMB)

**Figure 5:**
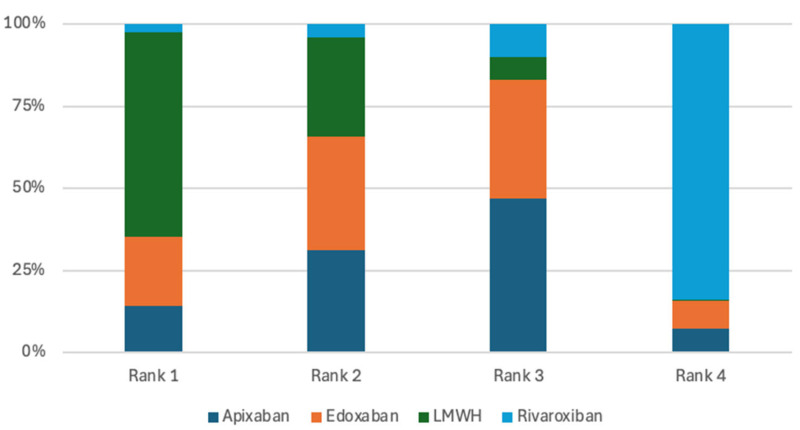
Treatments ranking for CRNMB.

The analysis of CRNMB included four studies [10,11,12,13]. In pairwise comparisons, LMWH did not demonstrate a lower risk of CRNMB when compared with apixaban, edoxaban or rivaroxaban, with odds ratios of 0.68 (95% CrI 0.23–2.07), 0.73 (95% CrI 0.17–3.10), and 0.24 (95% CrI 0.05–1.18), respectively. Similarly, apixaban did not reduce CRNMB risk relative to rivaroxaban (OR 0.36, 95% CrI 0.05–2.39) or edoxaban (OR 1.08, 95% CrI 0.17–6.46) (Table 4). Despite the absence of statistically significant differences, LMWH had the highest probability of being ranked first for minimizing CRNMB, with a 62.37% likelihood of occupying the top position (Figure 5). However, this finding is a probabilistic estimate and should be interpreted vigilantly due to non-significant treatment differences in the direct and indirect network comparisons.

### 3.5. Mortality

**Figure 6:**
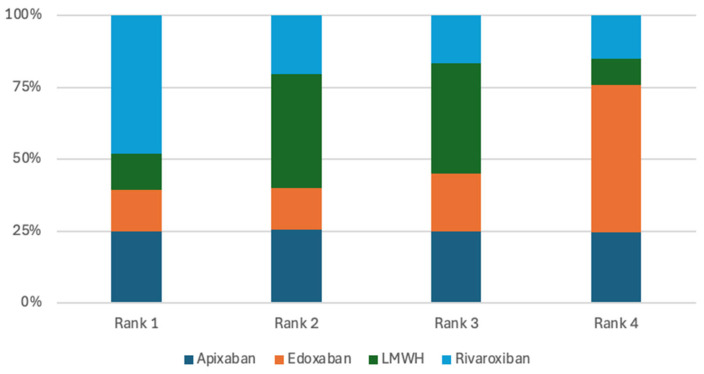
Treatments ranking for Mortality.

The mortality outcome analysis included six randomized clinical trials [8,9,10,11,12,13]. The pairwise comparisons of treatments showed no significant differences in reducing mortality risk (Table 5). However, the rivaroxaban treatment was ranked highest in terms of probability for lowering mortality risk, with a 48.1% likelihood of being the top treatment option (Figure 6). Rivaroxaban was associated with the highest probability of ranking first for mortality; however, this probable advantage is not a true treatment estimate and requires a careful interpretation of the findings due to no statistically significant differences across treatment strategies.

### 3.6. Sensitivity Analysis

To reduce outcome heterogeneity, two studies that assessed outcomes at time points different from the majority of randomized controlled trials—specifically at 3 months and 12 months, rather than the common 6-month follow-up—were excluded. Accordingly, Planquette et al. (2021) and Raskob et al. (2018) [12,13] were systematically removed from all analyses while preserving the original network structure. This sensitivity analysis showed no meaningful change in the results for VTE recurrence, major bleeding, clinically relevant non-major bleeding, or mortality [Supplementary Tables S1–S4].

## 4. Discussion

This network meta-analysis enables indirect head-to-head comparison of anticoagulant therapies for venous thromboembolism in patients with cancer. No significant differences were observed in VTE recurrence, major bleeding, clinically relevant non-major bleeding, or mortality among apixaban, rivaroxaban, edoxaban, LMWH, and warfarin, although major bleeding and CRNMB were significantly increased in edoxaban and rivaroxaban individual trials, respectively. Remarkably, warfarin contributed data from a limited number of patients derived mainly from subgroup analyses; therefore, definitive conclusions regarding its equivalence to other treatments should be interpreted with caution. In fact, several randomized controlled trials, including CLOT and CATCH, have demonstrated that LMWH is more effective than warfarin in reducing VTE recurrence and is associated with a lower incidence of clinically relevant non-major bleeding [18,19].

Although pairwise comparisons did not identify statistically significant differences, treatment ranking based on SUCRA probabilities suggested relative variation across outcomes. Apixaban ranked highest for VTE recurrence prevention and major bleeding, LMWH ranked highest for minimizing clinically relevant non-major bleeding, and rivaroxaban demonstrated the highest probability of reducing mortality. These rankings should be interpreted with caution, as they reflect probabilistic estimates rather than definitive treatment effects. The wide credible intervals observed across comparisons indicate substantial uncertainty, largely attributable to the limited number of included trials and low event rates.

The baseline characteristics of the patients across the seven randomized controlled trials (RCTs) included in the analysis were generally comparable, with minor differences observed in the proportions of metastatic disease, tumor types, and methods for evaluating primary and secondary outcomes, as outlined in the Supplemental Table S5. The CARAVAGGIO trial contributed the largest proportion of patients to the meta-analysis, comprising 1155 of 3325 participants (34.7%), followed by the Hokusai-VTE Cancer trial, which enrolled 1046 patients (31.5%) [9,13]. The percentage of metastatic patients varied from 33% to 84% across the trials, with the Caravaggio trial reporting 68%, which included patients with recurrent or locally advanced cancers [9].

All trials primarily included patients with various solid tumors, although specific details were lacking in some studies. For instance, the McBane et al. trial did not specify the tumor types, while the majority of patients (42 out of 100) in the Mokadem et al. study had colon cancer [8,9,10,11]. Additionally, concurrent systemic chemotherapy was reported in four studies (McBane 2020, Young 2018, Agnelli et al., 2020, and Raskob et al., 2018), where 62% to 83% of the participants received ongoing chemotherapy during the trial period [8,9,10,13]. The presence of variations in trial design, follow-up duration, cancer types, concurrent chemotherapy, and metastatic status can limit the precision of indirect comparisons.

The definition of major bleeding was consistent across the trials, utilizing the International Society on Thrombosis and Haemostasis (ISTH) criteria. VTE recurrence was uniformly defined as a new symptomatic deep vein thrombosis (DVT) or pulmonary embolism (PE) confirmed by imaging. However, the Caravaggio trial (Agnelli et al., 2020, Raskob et al., 2018, and Planquette et al., 2021) expanded this definition to include incidental VTE, which may have impacted the observed recurrence rates. Although the alignment of significant bleeding criteria enhances comparability across trials, variations in including incidental VTE necessitate careful interpretation when comparing the efficacy of different treatment regimens [9,12,13]. Planquette et al. (2021), [12] VTE recurrence was defined to include worsening of pulmonary or lower-limb vascular obstruction, a criterion that is not consistently used across clinical trials. This non-standard definition may have led to an overestimation of VTE recurrence and apparent treatment failure [12] (Supplemental Table S5).

Apixaban, rivaroxaban, and edoxaban demonstrated comparable efficacy to LMWH for preventing recurrent VTE, across RCTs. Differences among DOACs were more apparent in bleeding outcomes. In the SELECT-D trial, clinically relevant non-major bleeding was significantly more frequent among patients receiving rivaroxaban, with a hazard ratio for the six-month cumulative incidence of 3.76 (95% CI: 1.63–8.69) [10]. Similarly, edoxaban was associated with a statistically significant increase in major bleeding compared with LMWH (*p* < 0.05) [13]. In contrast, apixaban was not associated with an increased risk of major bleeding or clinically relevant non-major bleeding in three randomized controlled trials [7,8,11] (Table 6).

Supporting this interpretation, a large retrospective study including a substantial number of participants found that the incidence of major bleeding was higher with rivaroxaban than with dabigatran, despite both agents having a comparable elimination half-life of approximately 12 h. This finding suggests that differences in dosing frequency, rather than half-life alone, may contribute to bleeding risk. Once-daily dosing regimens are associated with greater peak–trough variability, which may lead to transient periods of intensified anticoagulant effect and thereby increase bleeding risk over time. In contrast, twice-daily agents such as apixaban and dabigatran provide more stable plasma concentrations throughout the dosing interval, potentially reducing the bleeding risk associated with peak anticoagulation exposure [20]. In addition, patient-specific factors, including concomitant drug–drug interactions, impaired renal function, and altered volume of distribution related to cancer-associated physiological changes, may further influence the safety profiles of direct oral anticoagulants.

Although apixaban, rivaroxaban, and edoxaban offer advantages over injectable unfractionated heparin (UFH) and low-molecular-weight heparin (LMWH) in terms of ease of administration and patient convenience, current clinical guidelines remain cautious in their recommendations. Recommendations from the American Society of Clinical Oncology and the National Comprehensive Cancer Network reflect the heterogeneity of bleeding risk across patient populations, particularly in those with gastric or gastroesophageal lesions [21,22].

The results of our network meta-analysis are generally consistent with those of the included randomized controlled trials in the prevention of VTE recurrence among apixaban, edoxaban, rivaroxaban, and LMWH. However, the network meta-analysis did not show a difference in major bleeding and CRNMB outcomes; one study showed increased risk of major bleeding in edoxaban, and another study showed increased risk of CRNMB in rivaroxaban versus LMWH. Therefore, clinicians should consider individual patient risk factors when choosing the anticoagulant in those high-risk populations [10,13].

Several limitations should be considered when interpreting the findings of this study. First, the network meta-analysis included a limited number of randomized controlled trials and low event rates, resulting in wide credible intervals for several indirect comparisons. Credible intervals represent a range in which the true value of the treatment effect would lie with a 95% probability. When the credible interval is wide, it reflects uncertainty and limited precision, and the corresponding treatment effect estimates should be interpreted cautiously. Second, the lack of individual patient-level data restricted the ability to perform subgroup analyses based on cancer type, metastatic status, or concurrent chemotherapy. Third, definitions of VTE recurrence were not fully consistent across trials, which may have affected the precision of outcome estimates. In addition, treatment duration and follow-up periods varied among studies; however, sensitivity analyses excluding trials with non-standard follow-up durations did not alter the efficacy or safety results. Finally, treatment rankings derived from SUCRA should be interpreted cautiously, as they reflect probabilistic estimates rather than definitive evidence of treatment superiority.

## 5. Conclusions

In this Bayesian network meta-analysis, apixaban, rivaroxaban, edoxaban, and LMWH demonstrated no statistically significant differences for VTE recurrence, major bleeding, CRNMB, or mortality. However, wide credible intervals for efficacy and safety outcomes should be interpreted cautiously due to uncertainty and limited precision.

## Figures and Tables

**Table 1:** Characteristics of the randomized controlled trials included in the network meta-analysis.

Study	Year	Study Design	Follow-Up	Patients (n)	Intervention	Comparator
Agnelli et al. [7]	2015	Randomized, double-blind trial	6 months	169	Apixaban 10 mg twice daily for 7 days, then 5 mg twice daily	Enoxaparin 1 mg/kg twice daily for ≥5 days, followed by dose-adjusted warfarin (INR 2–3)
McBane et al. [8]	2020	Randomized, open-label trial	6 months	287	Apixaban 10 mg twice daily for 7 days, then 5 mg twice daily	Dalteparin 200 IU/kg once daily for 1 month, then 150 IU/kg once daily
Agnelli et al. [9]	2020	Randomized, open-label, non-inferiority trial	6 months	1155	Apixaban 10 mg twice daily for 7 days, then 5 mg twice daily	Dalteparin 200 IU/kg once daily for 1 month, then 150 IU/kg once daily
Young et al. [10]	2018	Randomized, open-label trial	6 months	406	Rivaroxaban 15 mg twice daily for 3 weeks, then 20 mg once daily	Dalteparin 200 IU/kg once daily for 1 month, then 150 IU/kg once daily
Mokadem et al. [11]	2020	Randomized, single-blind trial	6 months	100	Apixaban 10 mg twice daily for 7 days, then 5 mg twice daily	Enoxaparin 1 mg/kg twice daily
Planquette et al. [12]	2021	Randomized, open-label, non-inferiority trial	3 months	158	Rivaroxaban 15 mg twice daily for 3 weeks, then 20 mg once daily	Dalteparin 200 IU/kg once daily for 1 month, then 150 IU/kg once daily
Raskob et al. [13]	2018	Randomized, open-label, non-inferiority trial	12 months	1046	Edoxaban 60 mg once daily after ≥5 days of LMWH	Dalteparin 200 IU/kg once daily for 1 month, then 150 IU/kg once daily

INR, international normalized ratio; IU, international units; LMWH, low-molecular-weight heparin.

**Table 2:** Pairwise comparison of columns with rows and presenting odds ratio (with 95% credible interval) from network meta-analysis for VTE recurrence outcome.

Treatment	Edoxaban	LMWH	Warfarin	Rivaroxaban
Apixaban	1.43 (0.15, 22.83)	2.18 (0.69, 11.18)	1.88 (0.16, 24.90)	1.13 (0.17, 12.27)
Edoxaban	—	1.50 (0.19, 12.36)	1.28 (0.03, 36.99)	0.77 (0.06, 11.11)
LMWH	—	—	0.85 (0.04, 12.92)	0.51 (0.10, 2.66)
Warfarin	—	—	—	0.60 (0.03, 19.00)

**Table 3:** Pairwise comparison of columns with rows and presenting the odds ratio (with 95% credible interval) from network meta-analysis for the primary bleeding outcome.

Treatment	Edoxaban	LMWH	Warfarin	Rivaroxaban
Apixaban	2.76 (0.34, 48.04)	1.58 (0.50, 9.73)	2.56 (0.20, 42.77)	1.88 (0.23, 20.88)
Edoxaban	—	0.56 (0.08, 4.14)	0.90 (0.02, 28.10)	0.66 (0.04, 7.63)
LMWH	—	—	1.56 (0.06, 30.41)	1.17 (0.18, 5.63)
Warfarin	—	—	—	0.73 (0.02, 25.03)

**Table 4:** Pairwise comparison of columns with rows and presenting odds ratio (with 95% credible interval) from network meta-analysis for CRNMB outcome.

Treatment	Edoxaban	LMWH	Rivaroxaban
Apixaban	0.93 (0.15, 5.83)	0.68 (0.23, 2.07)	2.81 (0.42, 20.19)
Edoxaban	—	0.73 (0.17, 3.1)	2.98 (0.35, 26.21)
LMWH	—	—	4.11 (0.85, 20.5)

**Table 5:** Pairwise comparison of columns with rows and presenting the odds ratio (with 95% credible interval) from network meta-analysis for the mortality outcome.

Treatment	Edoxaban	LMWH	Rivaroxaban
Apixaban	1.13 (0.48, 2.31)	0.99 (0.57, 1.54)	0.9 (0.41, 1.81)
Edoxaban	—	0.89 (0.48, 1.63)	0.8 (0.36, 1.85)
LMWH	—	—	0.9 (0.53, 1.59)

**Table 6:** Major bleeding and CRNMB in included studies.

Study	Total Patients (n)	Active Arm	Control Arm	Major Bleeding in Active Arm	Major Bleeding in Control Arm	CRNMB in Active Arm	CRNMB in Control Arm
Agnelli et al., 2015 [7]	169	Apixaban	Warfarin	2/87 (2.3%)	4/80 (5.0%)	N/A	N/A
McBane et al., 2020 [8]	287	Apixaban	Dalteparin	0/145 (0.0%)	2/142 (1.4%)	9/145 (6.2%)	7/142 (4.9%)
Agnelli et al., 2020 [9]	1155	Apixaban	Dalteparin	22/576 (3.8%)	23/579 (3.9%)	52/576 (9.0%)	35/579 (6.0%)
Young et al., 2018 [10]	406	Rivaroxaban	Dalteparin	11/203 (5.4%)	6/203 (2.9%)	25/203 (12.3%) *	7/203 (3.4%)
Mokadem et al., 2020 [11]	100	Apixaban	Enoxaparin	2/50 (4.0%)	4/50 (8.0%)	N/A	N/A
Planquette et al., 2021 [12]	158	Rivaroxaban	Dalteparin	1/74 (1.4%)	3/84 (3.6%)	N/A	N/A
Raskob et al., 2018 [13]	1046	Edoxaban	Dalteparin	36/522 (6.9%) †	21/524 (4.0%)	76/522 (14.6%)	58/524 (11.1%)

* Hazard ratio for 6-month cumulative incidence of CRNMB: 3.76 (95% CI, 1.63–8.69). † *p* < 0.05. CRNMB, clinically relevant non-major bleeding; N/A, not available. Note: Unless otherwise indicated, reported differences were not statistically significant.

## Data Availability

The authors confirm that the data supporting the findings of this study are available within the article.

## Supplementary Materials

The following supporting information can be downloaded at: https://www.mdpi.com/article/10.3390/jcm15031090/s1, File S1: PRISMA_2020_checklis, Ref. [23]; Table S1: Pairwise comparison of columns with rows and presenting odds ratio (with 95% credible interval) from network meta-analysis for VTE recurrence outcome; Table S2: Pairwise comparison of columns with rows and presenting the odds ratio (with 95% credible interval) from network meta-analysis for the major bleeding outcome; Table S3: Pairwise comparison of columns with rows and presenting odds ratio (with 95% credible interval) from network meta-analysis for CRNMB outcome; Table S4: Pairwise comparison of columns with rows and presenting the odds ratio (with 95% credible interval) from network meta-analysis for the mortality outcome; Table S5: Patient characteristics and definitions of outcomes in the included studies.
