# Supplementary material for: Safety and Efficacy of Direct Oral Anticoagulants Versus Standard Therapy for Venous Thromboembolism in Cancer Patients: A Systematic Review and Network Meta-Analysis of Randomized Clinical Trials
Source: J Clin Med. 2026 Jan 30;15(3):1090. doi: 10.3390/jcm15031090 (PMC12898735; doi:10.3390/jcm15031090)
Supplement: Supplementary file 1 [file jcm-15-01090-s001.zip › jcm-4095956-supplementary.pdf]

Supplemental Table S1: Pairwise comparison of columns with rows and presenting odds ratio (with 95% credible interval) from network meta-analysis for VTE recurrence outcome.

Treatments	Dalteparin	Warfarin	Rivaroxaban
Apixaban	2.31 (0.60, 13.26)	1.88 (0.12, 30.03)	0.93 (0.06, 20.77)
Dalteparin	-	0.79 (0.03, 16.29)	0.40 (0.03, 4.71)
Warfarin	-	-	0.51 (0.01, 31.85)

Supplemental Table S2: Pairwise comparison of columns with rows and presenting the odds ratio (with 95% credible interval) from network meta-analysis for the major bleeding outcome.

Treatments	Dalteparin	Warfarin	Rivaroxaban
Apixaban	1.60 (0.5, 9.83)	2.52 (0.19, 40.83)	3.18 (0.30, 59.78)
Dalteparin	-	1.54 (0.06, 28.6)	1.97 (0.22, 17.26)
Warfarin	-	-	1.27 (0.04, 64.63)

Supplemental Table S3: Pairwise comparison of columns with rows and presenting odds ratio (with 95% credible interval) from network meta-analysis for CRNMB outcome.

Treatments	Dalteparin	Rivaroxaban
Apixaban	0.68 (0.24, 2.11)	2.79 (0.42, 19.77)
Dalteparin	-	4.07 (0.83, 20.45)

Supplemental Table S4: Pairwise comparison of columns with rows and presenting the odds ratio (with 95% credible interval) from network meta-analysis for the mortality outcome.

Treatments	Dalteparin	Rivaroxaban
Apixaban	0.99 (0.55, 1.55)	0.79 (0.31, 1.85)
Dalteparin	-	0.81 (0.39, 1.66)

Supplemental Table S5: Patient characteristics and definitions of outcomes in the included studies.

Study	Total Patients (n)	Metastatic Cancer (%)	Most Common Cancer Type	Concurrent Chemotherapy (%)	Definition of VTE Recurrence	Definition of Bleeding Outcomes
**Agnelli et al., 2015** [7]	169	~33%	Variety of solid tumors	Not reported	New symptomatic DVT or PE confirmed by imaging	ISTH criteria for major bleeding
**McBane et al., 2020** [8]	287	64.3%	Not specified	72.6%	New symptomatic DVT or PE confirmed by imaging	ISTH criteria for major bleeding
**Agnelli et al., 2020** [9]	1155	68% (including recurrent locally advanced cancer)	Variety of solid tumors	62%	Symptomatic DVT or PE confirmed by imaging; incidental VTE included	ISTH criteria for major bleeding
**Young et al., 2018** [10]	406	58%	Variety of solid tumors	83%	New symptomatic DVT or PE confirmed by imaging	ISTH criteria for major bleeding
**Mokadem et al., 2020** [11]	100	84%	Colon cancer (42%)	Not reported	New symptomatic DVT or PE confirmed by imaging	ISTH criteria for major bleeding
**Planquette et al., 2021** [12]	158	72.8%	Variety of solid tumors	65.8%	Symptomatic DVT or PE confirmed by imaging; incidental VTE included; worsening pulmonary or lower-limb vascular obstruction	ISTH criteria for major bleeding
**Raskob et al., 2018** [13]	1046	52.9%	Variety of solid tumors	Not reported	Symptomatic DVT or PE confirmed by imaging; incidental VTE included	ISTH criteria for major bleeding

DVT, deep vein thrombosis; PE, pulmonary embolism; ISTH, International Society on Thrombosis and Haemostasis; VTE, venous thromboembolism.
